# Ethanol extract of *Paridis rhizoma* attenuates carrageenan-induced paw swelling in rats by inhibiting the production of inflammatory factors

**DOI:** 10.1186/s12906-023-04264-6

**Published:** 2023-12-04

**Authors:** Li Xiang, Qinwan Huang, Tao Chen, Qingman He, Huan Yao, Yongxiang Gao

**Affiliations:** 1 Hospital of Chengdu University of Traditional Chinese Medicine, Chengdu, 610072 Sichuan China; 2 School of Pharmacy, Cheng du University of Traditional Chinese Medicine, Chengdu, 610072 Sichuan China; 3 Sichuan Provincial Engineering Research Center of Innovative Re-Development of Famous Classical Formulas, Pengzhou, 611930 Sichuan China

**Keywords:** *Paridis rhizoma* extract, Paw edema, Inflammation, Reactive oxygen species, NF-kB pathway

## Abstract

**Context:**

Inflammation has been identified as a key factor contributing to the development of numerous diseases. Several anti-inflammatory drugs have been developed to treat inflammation-related diseases. However, some of such drugs are associated with varying degrees of side effects. Therefore, it is imperative to develop new anti-inflammatory drugs with reducing side effects for the treatment of inflammation-related diseases. Natural anti-inflammatory drugs have emerged as an important area of research in recent years. The study was to determine the anti-inflammatory mechanism of *Paridis rhizoma* extract (PRE) in rat models of acute inflammation induced by carrageenan and RAW264.7 cells models induced by lipopolysaccharide (LPS).

**Materials and methods:**

PRE was investigated using the carrageenan-induced paw oedema model on rats in vivo. Histopathology examined the extent of inflammatory infiltration and tissue damage. The effect of PRE on the levels of specific cytokines was determined using enzyme-linked immunosorbent assay (ELISA). The Cell Counting Kit (CCK)-8 assay evaluated the cytotoxic effects of PRE on Raw264.7 cells. The mRNA expression levels of cytokines were quantified using quantitative real-time reverse transcriptase polymerase chain reaction (RT-PCR). Western blot measured TNF-α, IL6, TLR4, p-P65, p-IKB, HO1, SOD1 and SOD2. Fluorescence measured the cellular levels of reactive oxygen species (ROS).

**Results:**

PRE treatment reduced interstitial edema and structural damage in a dose-dependent manner in vivo. PRE inhibited inflammatory responses in vivo and in vitro, as evidenced by the decreased expression of inflammatory factors, production of ROS, and increased expression of SOD1, SOD2, and HO1. Moreover, PRE inhibited the activity of the nuclear factor kappa B (NF-kB) pathway.

**Conclusion:**

The anti-inflammatory activity and potential mechanism of PRE were demonstrated according to the results. PRE reduced LPS-induced inflammation in RAW264.7 cells by inhibiting the NF-KB signaling pathway and ROS production in vitro. PRE alleviated interstitial edema and structural damage in the carrageenan-induced paw edema model on rats in vivo. This study provided an idea for future development of PR-based anti-inflammatory drugs.

**Supplementary Information:**

The online version contains supplementary material available at 10.1186/s12906-023-04264-6.

## Introduction

Inflammation is an important immunological response induced by harmful stimuli such as pathogens, toxins, injury, infection, and/or irritation [1]. Therefore, it is an important defensive mechanism in the body [2]. Inflammatory processes are driven by several factors including white blood cells, plasma, and fluid at the site of inflammation. Changes in levels of molecules released by immune defence cells and other signalling molecules such as histamine, prostaglandins, leukotrienes, oxygen- and nitrogen-derived free radicals, and serotonin contribute to the occurrence of inflammation [3].

In a previous study, carrageenan stimulation increased the release of major indicators of inflammation. This outcome was characterized by the production of several pro-inflammatory factors such as bradykinin, histamine, prostaglandin, thrombin, and reactive oxygen species (ROS). These factors were produced at the site of injury or a large number of infiltrating cells [4–6]. The interaction of inflammatory factors and mediators caused redness, swelling, fever, pain, and loss of tissue function which were typical symptoms of local inflammation [7].

LPS-stimulated macrophages produced pro-inflammatory cytokines, including the tumor necrosis factor-α (TNF-α), interleukin-6 (IL-6), and IL-1β, as well as pro-inflammatory mediators, such as, nitric oxide (NO), prostaglandin E2 (PGE2), and ROS [8–10]. Toll-like receptors (TLRs) were transmembrane proteins which were expressed in immune cells. The recognition of LPS by the extracellular N-terminal region of TLRs was considered a key process that mediated the induction of inflammatory response [11]. LPS-induced activation of TLR had been found to activate the nuclear factor-kappaB (NF-kB) signaling pathways [12], which then regulated the development and progression of inflammation [13], promoted the production of iNOS, cyclooxygenase (COX)-2, and pro-inflammatory cytokines (TNF-α, IL-1β, IL-6, and IL-8) [14].

Non-steroidal anti-inflammatory drugs (NSAIDs) are among the most commonly prescribed drugs for decreasing pain and inflammation. However, long-term use of NSAIDs may result in clinically severe complications of the gastrointestinal tract, cardiovascular, liver, kidney, brain, and lungs [15]. Therefore, the search for natural anti-inflammatory drugs with fewer side effects have become an important direction of research.

*Paridis Rhizoma (Chonglou)*, is the dried root and rhizome of *Paris polyphylla* var *yunnanensis* (Franch.) Hand. -Mazz, or *Paris polyphylla* Smith var. *chinensis* (Franch.) Hara of Lilaceae family. Phytochemical and pharmacological studies had shown that PR was rich in steroidal saponins with significant anti-cancer, anti-inflammatory, haemostasis, immune regulation, and antioxidant properties [16–18]. Qian confirmed that PRE had significant anti-tumor and anti-angiogenic effects in a mouse model [19]. Polyphyllin I (PPI), which was the main component of *Paridis Rhizoma*, inhibited vasculogenic mimicry formation in hepatocellular carcinoma lines and transplanted hepatocellular carcinoma cells [20]. At present, there are few researches on the anti-inflammatory mechanism of PR. In this study, the anti-inflammatory effect and potential mechanism of PR extract were investigated.

## Materials and methods

### Reagents

The H&E staining kit was purchased from BaSO (Zhuhai, China). Carrageenan was brought from Solarbio (Beijing, China).

### Preparation of PRE

PRE was generously donated by Dr Huang from the School of Pharmacy, Cheng du University of traditional Chinese medicine. The details were as follows: *Paridis rhizoma* was ground and sifted through a 65-mesh (250 μm) sieve. The resulting powder was weighed to approximately 0.5 g and placed in a conical bottle with a cork. Then, 25 mL of 70% ethanol was added to the bottle. The contents were heated and refluxed at a specific temperature for 30 min. After cooling, the bottle was weighed again, and the lost weight was compensated by adding 70% ethanol. The mixture was shaken thoroughly and centrifuged at 12,000 × g·min-1 for 15 min. The resulting supernatant was filtered through a 0.22 μm microporous filter membrane to obtain the PRE.

### Cell culture

RAW264.7 cells were purchased from Procell Life Science and Technology Co., Ltd. (Wuhan, China) and incubated at 37 °C in 5% CO2 humidified air in Dulbecco’s Modified Eagle Medium (DMEM; Gibco, USA) supplemented with a mixture of 1% penicillin/streptomycin and 10% fetal bovine serum (FBS).

### Cell viability assay

The Cell Counting Kit (CCK)-8 assay (Biosharp, China) was used to evaluate the cytotoxic effects of PRE on Raw264.7 cells. Briefly, the cells were seeded into 96-well plates at a density of 4 × 10^3^ /ml and cultured for 24 h at 37 °C in an atmosphere of 5% CO2.The cells were then treated with different concentrations of PRE (0, 10, 25, 50, 100, 200, 400, 800 ng/ml) for 24 h. The viability of cells after the treatments was evaluated by addition of 10μL of CCK-8 solution (Beyotime, Shanghai, China) to each well and incubated at 37 °C for 2 h. The final absorbance was read at a wavelength of 450 nm using a microplate reader (Molecular Device, Shanghai, China).

### Western blotting

Protein was isolated from cells using a radioimmunoprecipitation assay buffer and quantified using a Bicinchoninic Acid Protein Assay Kit (Beyotime, Jiangsu, China). The proteins were separated using 10% sodium-dodecyl-sulfate polyacrylamide gel electrophoresis (SDS-PAGE) and transferred onto polyvinylidene difluoride membranes. The membranes were blocked with 5% skimmed milk in Tris-buffered saline for 1 h and were incubated with anti-phospho-p65 (Affinity, USA), anti-p65 (Affinity, USA), anti-phospho-IKB alpha (Affinity, USA), anti-IKB alpha (Affinity, USA), anti-IL-6 (Proteintech), anti-TNF alpha (Proteintech), and anti-β-actin (Proteintech) at 4 °C overnight. They were then washed with Tris-buffered saline Tween-20, and incubated with horseradish peroxidase-conjugated secondary antibody for 1 h at room temperature. Proteins blots were detected with enhanced chemiluminescence reagent (Beyotime, Jiangsu, China).

### Quantitative real-time reverse transcriptase polymerase chain reaction

Total RNA was isolated from cells using TRIzol™ (Solarbio, Beijing, China) reagent according to the manufacturer's instructions. The cDNA was synthesized using reverse transcription kit of the first strand using the SynScript® III cDNA Synthesis Mix. The mRNA levels were analysed by qRT-PCR (Bio-ER) in a total volume of 20μL using 2 × TSINGKE® Master qPCR Mix (SYBR Green I, Tsingke, China). Relative mRNA expression was calculated using the 2^‑ΔΔct^ method after normalization to the expression level of GAPDH expression. The gene-specific primers used are listed in the Table 1.

**Table 1:** Primers used for qPCR

	Forward (5′-3′)	Reverse ((5′-3′)
TNF-α	CCCTCACACTCAGATCATCTTCT	GCTACGACGTGGGCTACAG
COX2	TGTGACTGTACCCGGACTGG	TGCACATTGTAAGTAGGTGGAC
PGE2	CGCGGTGGCTGTCATCA	AGGGTTGGGTCCCAGGAAT
IL-6	GGATACCACTCCCAACAGACC	TTCTGCAAGTGCATCATCGT
CD86	ATGGACCCCAGATGCACCAT	CGGCAGATATGCAGTCCCAT
GAPDH	CCCTTCATTGACCTCAACTACATGG	CATGGTGGTGAAGACGCCAG

### Measurement of reactive oxygen species

RAW264.7 cells were cultured overnight in 6-well plates at a density of 1 × 10^6^ cells/well and then exposed to different concentrations of PRE and LPS (200 ng/ml) alone or in combination. The intracellular ROS level in Raw264.7 cells was determined using 2′,7′-Dichlorodihydrofluorescein diacetate (DCFH-DA; Yeasen). Simply, Raw264.7 cells were incubated with 10 µM DCFH-DA in DMEM without FBS at 37 °C for 20 min and washed three times with DMEM. The 2′,7′-dichlorofluorescein (DCF) fluorescence was observed using an inverted fluorescence microscope. The average fluorescence intensity was assessed using ImageJ.

### Animals

Adult male Sprague–Dawley rats (8–10 weeks old, 180–200 g) were purchased from SPF Laboratory Animal Technology Co., Ltd. (Beijing, China) and acclimatized for 1 week before experimentation. All animal procedures were performed in compliance with the National Institutes of Health Guidelines for Care and Use of Laboratory Animals. The protocol for this study was approved by the Bioethics Committee of Chengdu University of Traditional Chinese Medicine (2022–65). 

### Carrageenan-induced rat paw edema model

According to Winter et al. [21], the paw edema was induced by carrageenan. Rats were randomly divided into 6 groups each comprising 10 rats. They were pretreated orally for a week with the vehicle (0.9% saline, control group, *n* = 10), model (untreated group, *n* = 10), PRE low dosage (PRE-LD) (low dose, *n* = 10), PRE medium dosage (PRE-MD) (medium dose, *n* = 10), PRE high dose (PRE-HD) (high dose, *n* = 10) and indomethacin (INDO) (5 mg/kg, po) [22]. Rats in the low-, medium-, and high-dose PRE groups received 0.315 g, 0.63 g, and 1.26 g/kg PRE daily, respectively. Edema was induced 30 min after the last treatment, by injection of 0.1 mL of carrageenan (100 µg/paw) in saline into the right hind paw while the control received 0.9% saline (0.1 ml). Inflammation was quantified by measuring the volume (mL) displaced by the paw using plethysmometer (Tai Meng Software Co., Ltd, Chengdu, China) at 0, 0.5, 1, 1.5, and 2 h after carrageenan injection. All rats were anesthetized by intraperitoneal injection of 3% pentobarbital sodium (50 mg/kg) [23].

### Histopathological examination

The paws were fixed overnight in 4% paraformaldehyde containing in 0.1 M PBS and embedded in paraffin. Slides with a 5-μm thickness were prepared, deparaffinized and stained with Haematoxylin/eosin (H&E).

### Enzyme-linked immunosorbent assay (ELISA)

The concentrations of tumor necrosis factor-α (TNF-α) (Jianglai Biotechnology, JL13202), prostaglandin E2 (PGE2) (Jianglai Biotechnology, JL12636), cyclooxygenase-2 (COX-2) (Jianglai Biotechnology, JL21044) and interleukin-6 (IL-6) (Jianglai Biotechnology, JL20896) in sera were detected using ELISA following the manufacturer’s instructions.

### Statistical analysis

The statistical analyses were performed with GraphPad prism 8.0. Data were presented as means ± standard error of the mean (SEM). Comparisons among multiple groups were performed by a one-way analysis of variance (ANOVA). If one-way ANOVA yielded a significant F ratio, Bonferroni post-hoc analysis or Tamhane's T2 was conducted between the groups. *P* values < 0.05 were considered statistically significant.

## Result

### The effect of PRE on carrageenan-induced paw edema in rats

**Fig. 1:**
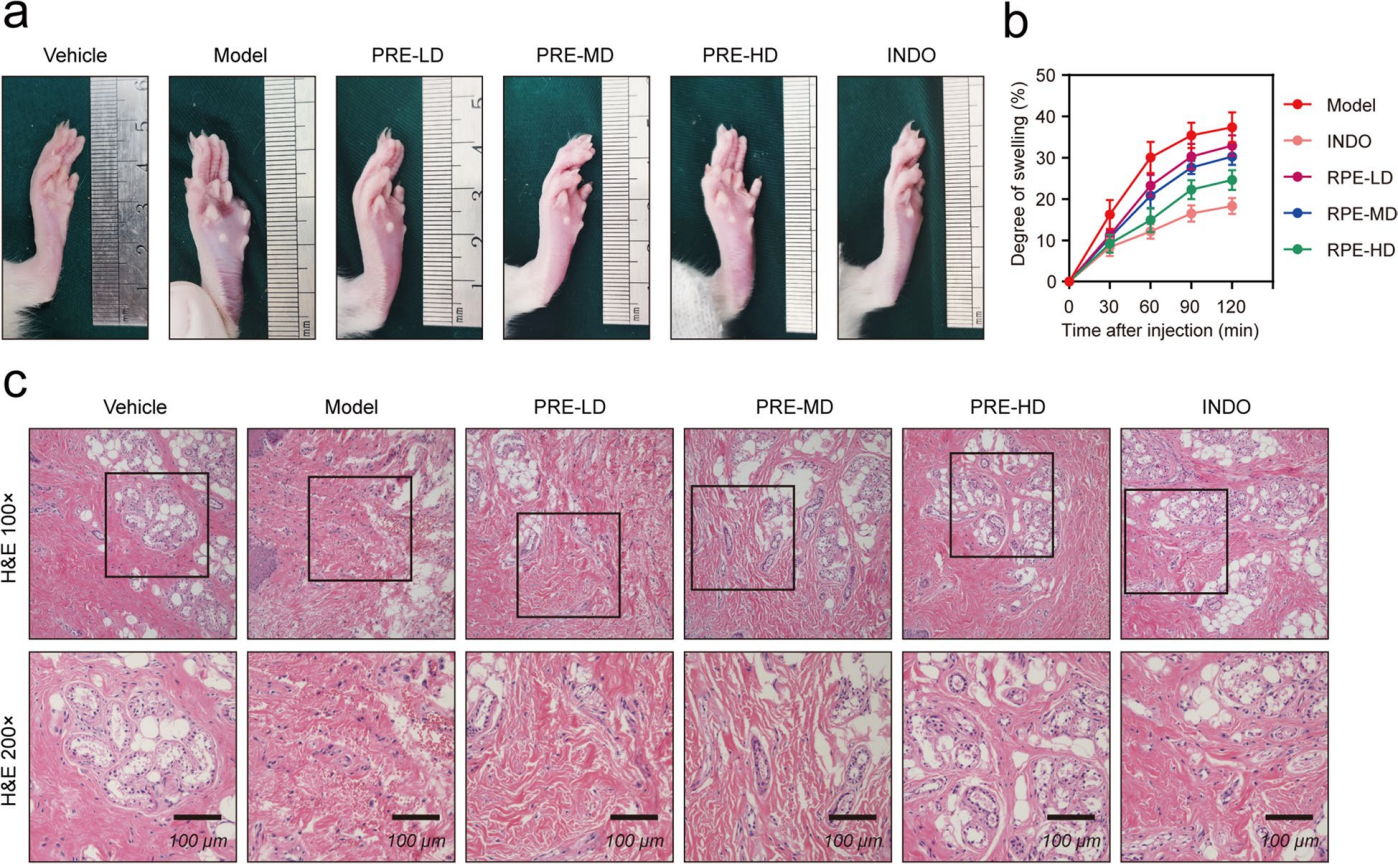
Effect of PRE on paw edema induced by carrageenan in rats. **a** Representative images of foot swelling in each group 120 min after modeling. **b** Quantitative statistics of foot swelling in each group at 30, 60, 90, and 120 min after modeling. **c** Representative image of H&E staining of rat paw. Data are presented as the mean ± SEM (*n* = 10). *p < 0.05, **p < 0.01

The injection of carrageenan into the sub-plantar region of the right hind paw rapidly induced paw edema. After 120 min of modeling, the right hind paws of rats in each group showed varying degrees of edema (Fig. 1a). Compared with the model group, PRE treatment reduced paw edema in a dose-dependent manner. The INDO group reduced foot edema significantly compared with the model group. And the INDO group showed lower foot swelling compared with the PRE-HD. These results suggested that PRE alleviated the foot swelling effects induced by carrageenan in rats. Further histopathological analysis indicated that the plantar tissue structure of the vehicle group was perfect and orderly, without visible inflammation, edema, bleeding, and tissue injury (Fig. 1c). Histopathologic symptoms such as interstitial edema, inflammatory exudation, vascular injury, massive exudation of red blood cells, and tissue organization disorder were observed in the model group. Compared with the model group, the aforementioned pathological changes were improved in the low-dose and medium-dose PRE groups. The PRE-HD and INDO groups had significantly reduced interstitial edema and structural damage. These results showed that PRE reduced edema, haemorrhages and inflammatory infiltration in rat plantar tissue induced by carrageenan.

### Effect of PRE on the expression of inflammatory cytokines in paw edema rats

**Fig. 2:**
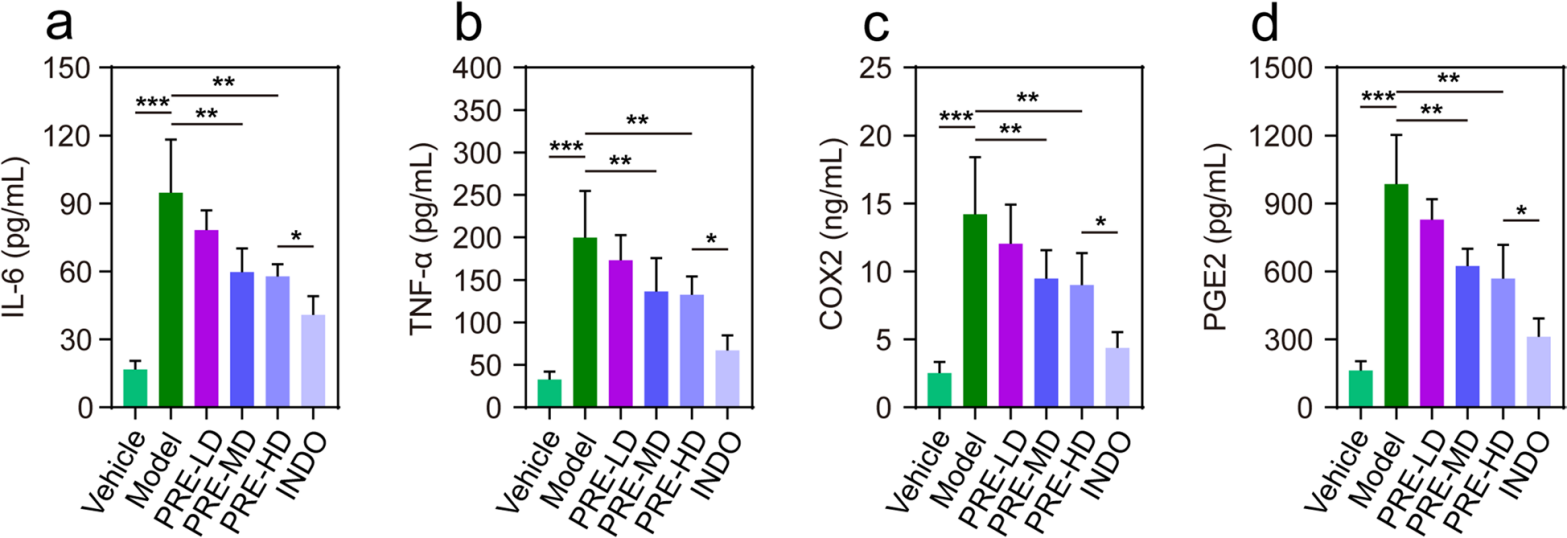
Effect of PRE on the expression of inflammatory cytokines in rats with foot swelling. **a**-**d** Serum levels of IL-6, TNF-α, COX2 and PGE2 in the indicated group. Data are presented as the mean ± SEM (*n* = 3). *p < 0.05, **p < 0.01, ***p < 0.001

The results of ELISA showed the levels of inflammatory cytokines IL-6, TNF-α, PGE2, and COX2 (Fig. 2a-d). Compared with the vehicle group, the above indexes were significantly higher in the model group (*P* < 0.001). PRE-MD and PRE-HD had lower levels of IL-6, TNF-α, COX2, and PGE2 compared with the Model group (*P* < 0.01). The levels of IL-6, TNF-α, PGE2, and COX2 in the INDO group were significantly lower compared with levels in the model group (*P* < 0.01), and the levels of IL-6, TNF-α, PGE2 and COX2 in the INDO group were better than those in the PRE-HD group (*P* < 0.05). These results revealed that PRE inhibited acute inflammation induced by carrageenan in rats.

### Effect of PRE on the expression of inflammatory cytokines in Raw264.7 cells induced by LPS

**Fig. 3:**
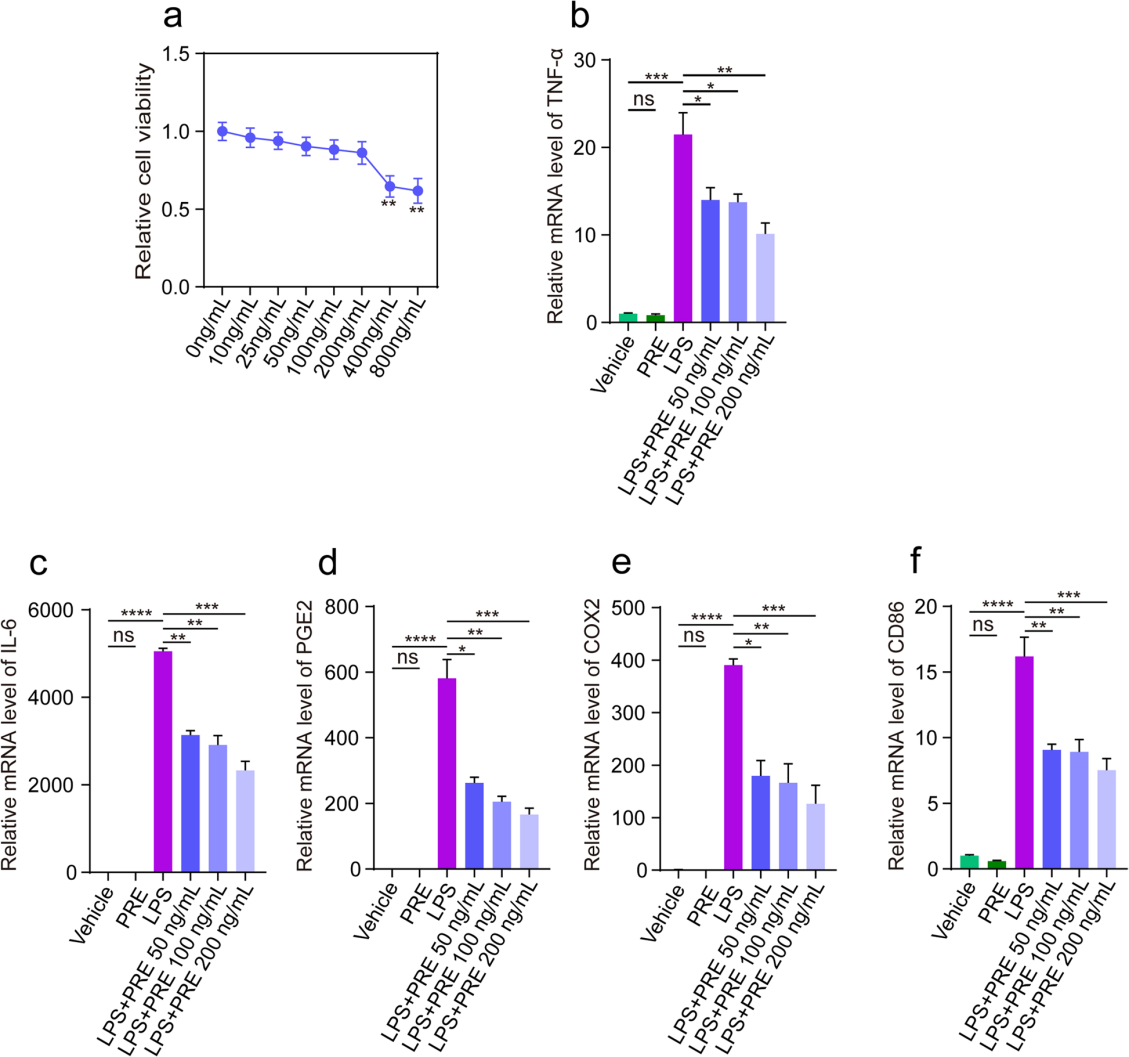
Effect of PRE on the expression of inflammatory cytokines in LPS-treated Raw264.7 cell line. **a** The viability of RAW264.7 cells was determined by the CCK-8 assay. Values of the treatment groups were normalized to those of the control group (representing 100% cell viability). **b**-**e** Relative mRNA expression of TNF-ɑ, COX2, PGE2, and IL-6. **f** The CD86 mRNA level of M1 phenotype. Data are presented as the mean ± SEM (*n* = 3). *p < 0.05, **p < 0.01, ***p < 0.001

The effect of PRE was evaluated using the monocyte-macrophage Raw264.7 cell line induced by LPS. The optimal PRE concentration was determined using the CCK-8 assay (Fig. 3a). Treatment with 400 ng/mL PRE caused significant cytotoxicity on RAW264.7 cells. Cells were exposed to a range of PRE concentrations (50 to 200 ng/mL). RT-PCR assay showed that PRE group (without LPS) had no effect on mRNA transcription of inflammatory cytokines, including TNF-α, COX2, PGE2, and IL-6 (Fig. 3b-e). However, PRE inhibited the expression of these genes in a dose-dependent manner after LPS (200 ng/mL) [24] treatment. This confirmed the inhibitory effect of PRE against LPS-induced inflammation. RT-PCR results revealed that the changes in CD86 were consistent with those of the above inflammatory factors. Macrophages are the critical effectors and regulators of inflammation. Pathogenic stimuli have been shown to activate macrophages. Based on these findings, we concluded that PRE inhibited pro-inflammatory activation and lowed the expression of inflammatory cytokines and mediators.

### Inflammatory response is related to oxidative stress

**Fig. 4:**
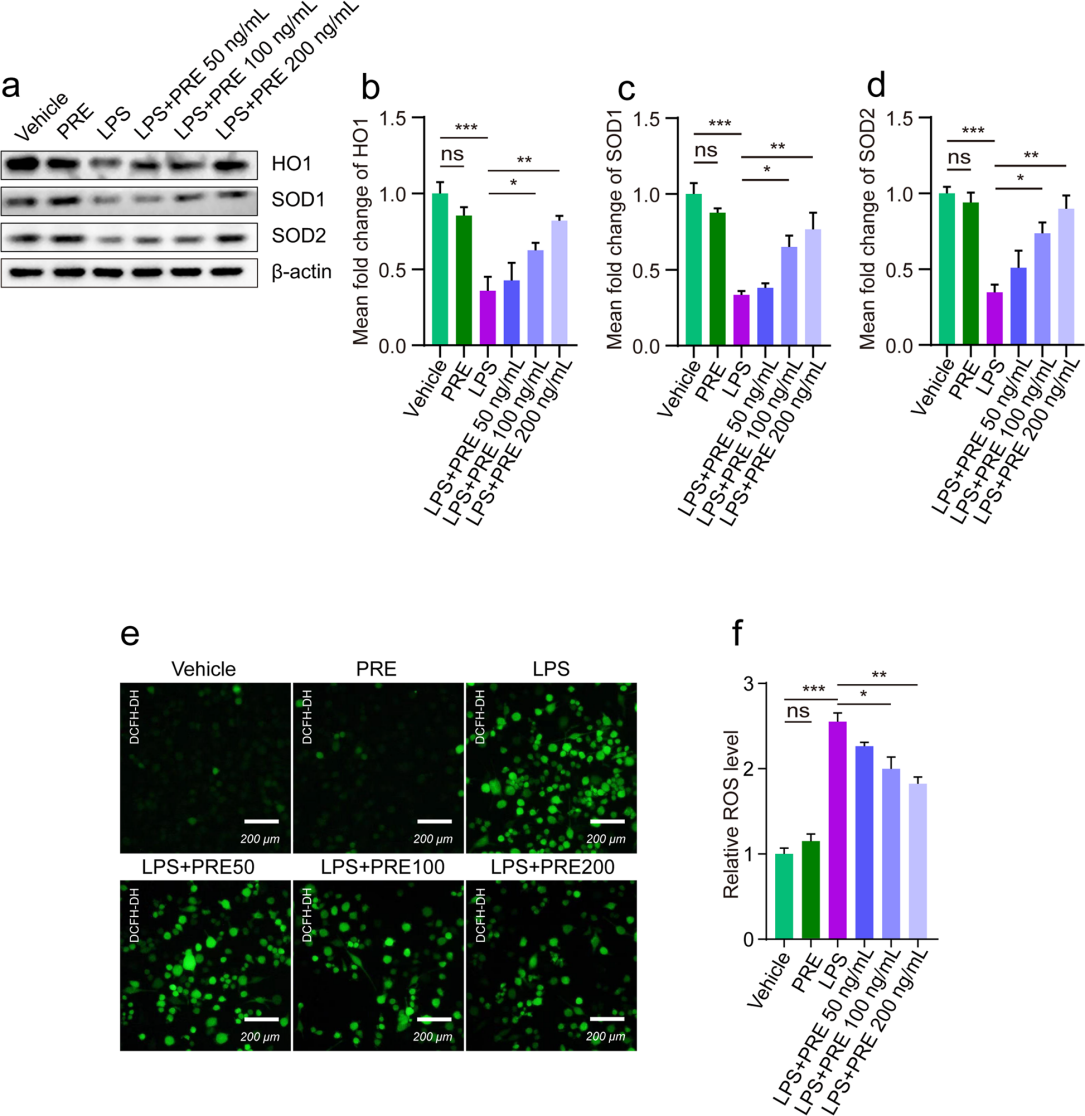
Effects of PRE on oxidative stress level in Raw264.7 cells treated with LPS. **a** Representative Western blot images showing changes in SOD1, SOD2, and HO1 protein levels in cells. **b**-**d** Quantification of HO1, SOD1, and SOD2 levels using ImageJ. **e** Representative DCFH-DA images of Raw264.7 cells treated with 200 ng/mL LPS for 12 h. **F** Relative ROS levels as quantified using ImageJ software. Data are presented as the mean ± SEM (*n* = 3). *p < 0.05, **p < 0.01, ***p < 0.001

Raw264.7 cells were activated by LPS to induce inflammatory response in *vitro*. Western blotting showed variations in the levels of SOD1, SOD2, and HO1 proteins in Raw264.7 cells (Fig. 4a). LPS inhibited the expression of SOD1, SOD2, and HO1, but those expression levels increased in a dose-dependent manner after PRE treatment (Fig. 4b-d). Immunofluorescence results corroborated these findings. After treatment of Raw264.7 cells with LPS, mean DCFH-DA fluorescence intensity increased significantly and decreased in a dose-dependent manner after PRE treatment (Fig. 4e-f). Therefore, these observations demonstrated that LPS induced oxidative stress damage leading to inflammation, and PRE exerted a therapeutic effect.

### PRE suppressed NF-kB pathway activation in *vitro*

**Fig. 5:**
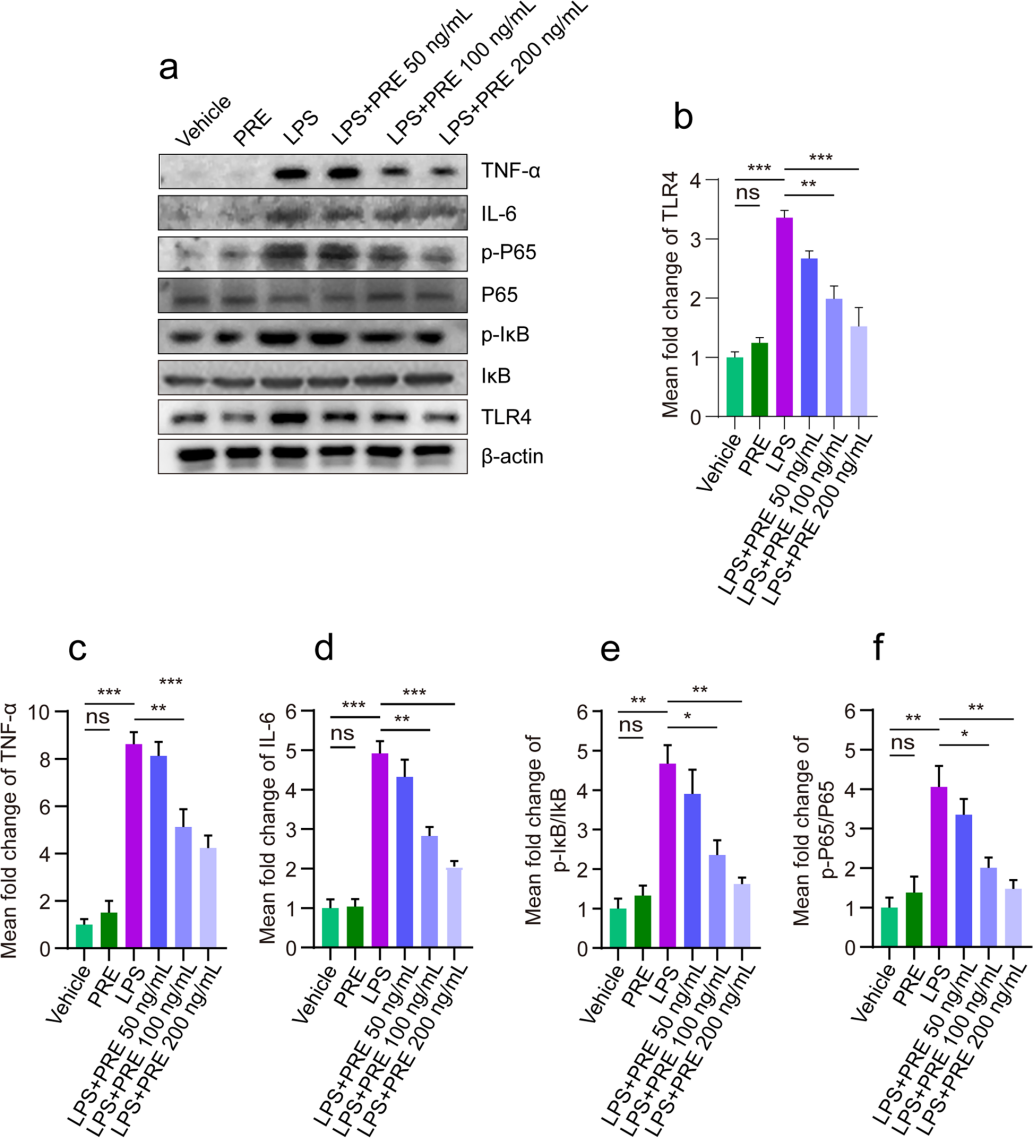
PRE inhibited the activation of the NF-kB pathway in vitro. **a** Representative western blotting images showing TNF-ɑ, IL-6, p-IKB, IKB, p-P65, P65 and TLR4 expression levels. **b**-**d** Quantification of TLR4, TNF-α, and IL-6 by ImageJ. **e **The mean fold change of p-IKB/IKB ratio. **f **The mean fold change of p-P65/P65 ratio. Data are presented as the mean ± SEM (*n* = 3). *p < 0.05, **p < 0.01, ***p < 0.001

To investigate the mechanism of PRE inhibiting acute inflammation, we examined the effect of PRE on the NF-kB pathway. The upstream signal of NF-kB, TLR4, was activated after LPS treatment with the release of inflammatory cytokines (Fig. 5a). Compared with the Vehicle and PRE (without LPS treatment) groups, LPS-treated cells had significantly higher levels of TLR4 (Fig. 5b). Thus, we explored the effect of PRE on the phosphorylation of NF-kB p65 and IKBα in LPs-stimulated RAW264.7 cells. LPS stimulation induced NF-kB p65 phosphorylation, which increased its transactivation potential [25]. PRE had no effect on the overall NF-kB p65 protein level, but decreased its phosphorylation level (Fig. 5e-f). In addition, PRE significantly inhibited LPS-induced phosphorylation of IKBα. These results suggested that PRE inhibited the phosphorylation of NF-kB and IKBα, hence sequestering NF-kB in the cytoplasm and inhibiting it transactivation potential. Therefore, PRE might down-regulate inflammatory response by inhibiting NF-kB activation in RAW264.7 cells stimulated by LPS. We confirmed that PRE treatment inhibited the activation of inflammation-related signaling pathways to attenuate inflammation in *vitro*.

## Discussion

Steroidal and non-steroidal anti-inflammatory drugs are currently used to treat inflammation-related diseases. However, several side effects and complications associated with such drugs had been reported [26]. The search for natural and plant-derived anti-inflammatory drugs emerged as an important area of research in recent years. Since inflammation was involved in the onset and progression of many diseases, targeting the inflammatory response period could yield good treatment outcomes. Various inflammatory mediators, such as PGE2, TNF-α and IL-6, were involved in the development of inflammatory diseases [9]. Thus, effectively inhibiting the production of these pro-inflammatory mediators might prevent the initiation and progression of inflammatory diseases. In this study, PRE significantly inhibited acute inflammation induced by carrageenan. Carrageenan-induced inflammation was characterized by increased polymorphonuclear neutrophil leukocyte infiltration and prostaglandin production, leading to the release of histamine, serotonin and bradykinin [27].

Prostaglandin E2 (PGE2) was a major physiologically active lipid that was synthesized from arachidonic acid by the cyclooxygenases (COX-1 and COX-2) and PGE synthases. PGE2 did not readily circulate throughout the body, and its activity was limited to the cells near the PGE2-producing cells [28]. Our results demonstrated that PRE reduced edema in rat paws (Fig. 1a-b). Thus, we postulated that local edema might be associated with the release of PGE2. Furthermore, the results of ELISA (Fig. 2) and pathology (Fig. 1c) revealed that inflammation was regulated by several cytokines, which were the key determinants of the cellular structure at the site of injury and modulate systemic responses to inflammation [3, 29, 30]. Compared with the model group, PRE treatment groups reduced tissue concentrations of IL-6 and TNF-α, and this effect was dose-dependent. Related studies had shown that IL-6 and TNF-α play crucial roles in the regulation of inflammatory response, including the activation of vascular endothelial cells, edema formation, inflammatory pain, and increased expression of specific chemokines and adhesion molecules. As a result, they promoted the direct recruitment of leukocytes to the site of inflammation [31–33].

LPS, as a stimulus that caused inflammatory responses in vivo and in vitro by activating the blinding of nuclear factor-kB (NF-kB) to the cell surface receptor toll-like receptor 4 (TLR4) [34]. This in turn enhanced the production of multiple inflammatory cytokines, thereby aggravating inflammation. Incubation of LPS with Raw264.7 cells increased the production of inflammatory cytokines and mediators (Fig. 3). Compared to the model group, treatment groups significantly reduced the levels of IL6 and TNF-ɑ. M1 macrophages were activated in vitro via the induction of LPS and IFN-γ to mimic the real environment [35]. Activation of macrophages by LPS triggered rapid polarization towards the M1 phenotype which secreted large amounts of pro-inflammatory cytokines and chemokines, and recruited more circulating neutrophils, monocytes, and mast cells such as IL-1β, IL-6, TNF-α, IL-12, ROS and inducible nitric oxide synthase (iNOS) [36, 37]. In this study, CD86 was significantly increased in LPs-stimulated macrophages (Fig. 3f), suggesting that the phenotypic shifted from M0 to M1 macrophage polarization characterizing the transition from normal to inflammation state. The inflammatory response mediated by toll-like receptor agonists might be linked to changes in macrophage phenotype. TLR4 was the principal sensor of LPS-induced macrophage-mediated inflammatory response. It was the only TLR that signalled through the myeloid differentiation primary reactive Protein 88 (MyD88) and TRIF signalling pathway to activate NF- kB and nuclear translocation to induce inflammation [36]. TLR4 overexpression activated the downstream NF-kB pathway (Fig. 5b). Before activation, NF-kB bound to IKBα, a cytoplasmic inhibitor of NF-kB. In LPS-stimulated macrophages, IKBα was phosphorylated by the IKBα kinase IKK and was degraded independently from NF-kB via the ubiquitin–proteasome pathway. The phosphorylation levels of several key signalling molecules of the NF-kB pathway was detected by Western blot to determine whether the LPS-induced polarization of macrophage M1 affected the activation of the NF-kB pathway. The results showed that PRE reduced the TNF-α, and IL-6 levels (Fig. 5c, d) and suppressed the phosphorylation levels of p65 and IKBα in LPS treated cells (Fig. 5e, f).

The level of ROS influenced the progression of many inflammatory diseases. It functioned as both a signalling molecule and an inflammatory mediator. It was produced by cells involved in the host defence response [38]. Inflammation and macrophage infiltration were linked to increased production of ROS [39]. LPS stimulation could promote the production of ROS in macrophages [40], and ROS could act as a secondary messenger that regulated the expression of pro-inflammatory cytokines [41]. Our results showed that PRE reduced ROS accumulation in LPS-treated RAW264.7 cells (Fig. 4e, f). In addition, antioxidant genes were significantly reduced following LPS treatment, whereas PRE treatment reversed this effect (Fig. 4a-d). Finally, PRE alleviated the LPS-induced oxidative damage in RAW264.7 cells and was thus a potential agent for preventing inflammatory responses by reducing ROS levels.

## Conclusion

In this study, PRE was evaluated the amelioration of inflammatory effects and could play anti-inflammatory role by affecting the NF-kB signaling pathways. As a natural anti-inflammatory drug, *Paridis rhizoma*, may be of great significance in the exploration of anti-inflammatory. The native potential drugs with the least side effects could be suitable to improve the health quality of patients.

### Supplementary Information

**Additional file 1: **Gray value of target protein bands (Groups from left to right).

## Data Availability

All data and results of the current study are available from the corresponding authors upon reasonable request. The original contributions presented in the study are included in the article.

## Abbreviations

PRE: Paridis rhizoma extract
PPI: Polyphyllin I
PRS: Paridis Rhizoma saponins
LPS: Lipopolysaccharide
ELISA: Enzyme-linked immunosorbent assay
RT-PCR: Quantitative real-time reverse transcriptase polymerase chain reaction
ROS: Reactive oxygen species
NF-KB: Nuclear factor kappa B
NSAIDs: Non-steroidal anti-inflammatory drugs
FBS: Fetal bovine serum
SDS-PAGE: Sodium-dodecyl-sulfate polyacrylamide gel electrophoresis
DCFH-DA: 2′,7′-Dichlorodihydrofluorescein diacetate
TLR4: Toll-like receptor 4
INDO: Indomethacin
H&E: Haematoxylin/eosin
