# Supplementary material for: Ethanol extract of Paridis rhizoma attenuates carrageenan-induced paw swelling in rats by inhibiting the production of inflammatory factors
Source: BMC Complement Med Ther. 2023 Dec 4;23:437. doi: 10.1186/s12906-023-04264-6 (PMC10696755; doi:10.1186/s12906-023-04264-6)
Supplement: Supplementary file 1 — Additional file 1: Gray value of target protein bands (Groups from left to right). [file 12906_2023_4264_MOESM1_ESM.pdf]

Supplementary Table

Gray value of target protein bands (Groups from left to right):

vehicle PRE LPS LPS+PRE 50ng/mL LPS+PRE 100ng/mL LPS+PRE 200ng/mL

The following gels corresponded to Figure5

Protein name:

IKB

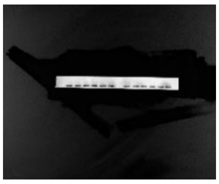

The right band

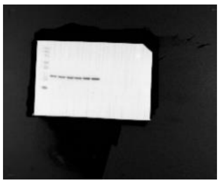

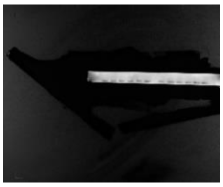

The left band

p-IKB

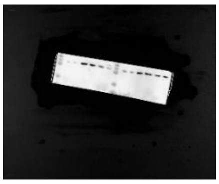

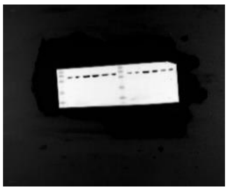

The left band

P65

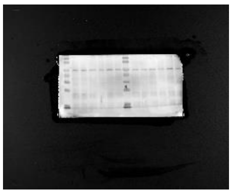

The right band

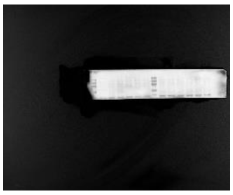

p-P65

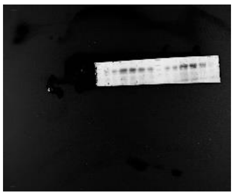

The left band

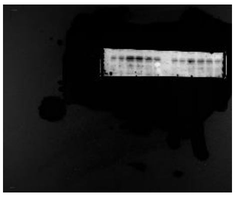

The left band

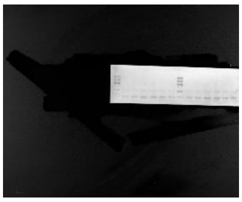

The right band

IL-6

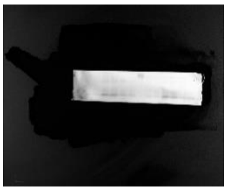

The left band

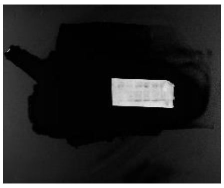

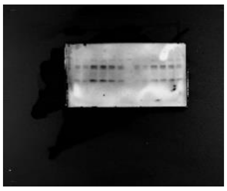

The left band

TNF-α

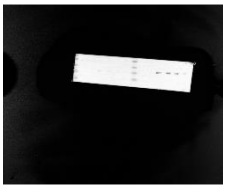

The right band

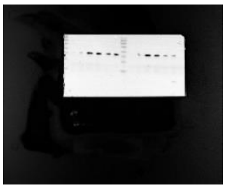

The right band

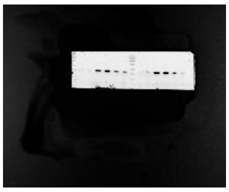

The left band

TLR4

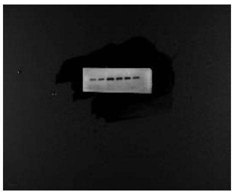

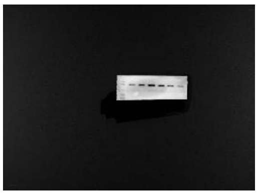

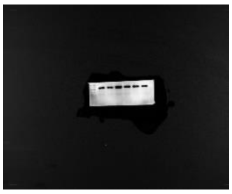

β-actin

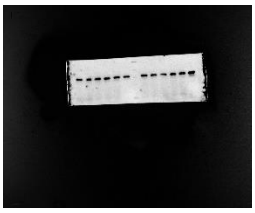

The left band

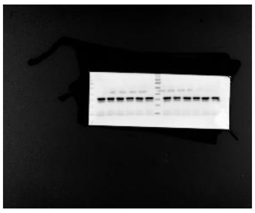

The following gels corresponded to Figure 4.

Protein name:

HO1

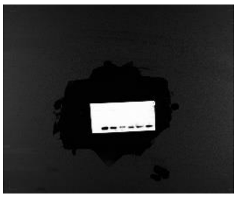

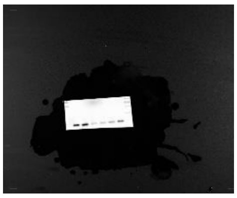

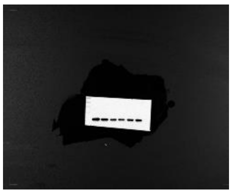

SOD1

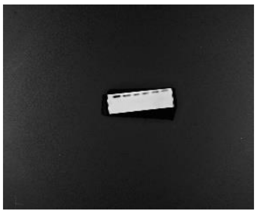
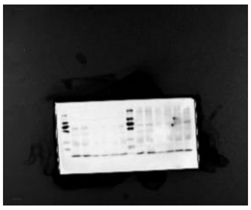
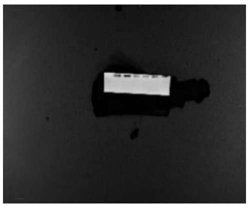

The left band

SOD2

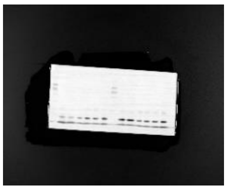
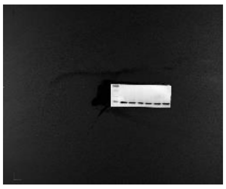
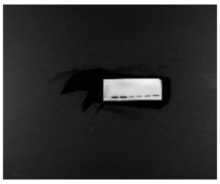

The right band

β-actin

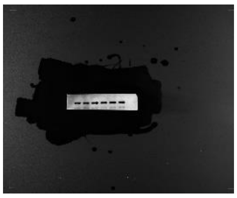
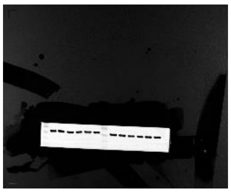
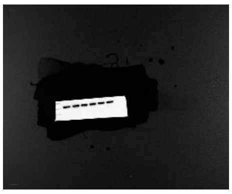

The left band
